# The Effect of the Topmost Layer and the Type of Bone Morphogenetic Protein-2 Immobilization on the Mesenchymal Stem Cell Response

**DOI:** 10.3390/ijms23169287

**Published:** 2022-08-18

**Authors:** Magdalena Wytrwal-Sarna, Małgorzata Sekuła-Stryjewska, Agata Pomorska, Ewa Ocłoń, Katarzyna Gajos, Michal Sarna, Ewa Zuba-Surma, Andrzej Bernasik, Krzysztof Szczubiałka

**Affiliations:** 1 Academic Centre for Materials and Nanotechnology, AGH University of Science and Technology, Al. A. Mickiewicza 30, 30-059 Krakow, Poland; 2 Malopolska Centre of Biotechnology, Jagiellonian University, Gronostajowa 7A, 30-387 Krakow, Poland; 3 Jerzy Haber Institute of Catalysis and Surface Chemistry, Polish Academy of Sciences, Niezapominajek 8, 30-239 Krakow, Poland; 4 Laboratory of Recombinant Proteins Production, Centre for Experimental and Innovative Medicine, University of Agriculture in Krakow, 1C Redzina Street, 30-248 Krakow, Poland; 5 Marian Smoluchowski Institute of Physics, Jagiellonian University, Stanislawa Łojasiewicza 11, 30-348 Krakow, Poland; 6 Faculty of Biochemistry, Biophysics and Biotechnology, Jagiellonian University, Gronostajowa 7, 30-387 Krakow, Poland; 7 Faculty of Physics and Applied Computer Science, AGH University of Science and Technology, Reymonta 19, 30-059 Krakow, Poland; 8 Faculty of Chemistry, Jagiellonian University, Gronostajowa 2, 30-387 Krakow, Poland

**Keywords:** diazoresin, chondroitin sulfate, bone morphogenetic protein 2, TOF-SIMS, AR-XPS, cell culture surfaces

## Abstract

Recombinant human bone morphogenetic protein-2 (rhBMP-2) plays a key role in the stem cell response, not only via its influence on osteogenesis, but also on cellular adhesion, migration, and proliferation. However, when applied clinically, its supra-physiological levels cause many adverse effects. Therefore, there is a need to concomitantly retain the biological activity of BMP-2 and reduce its doses. Currently, the most promising strategies involve site-specific and site-directed immobilization of rhBMP-2. This work investigated the covalent and electrostatic binding of rhBMP-2 to ultrathin-multilayers with chondroitin sulfate (CS) or diazoresin (DR) as the topmost layer. Angle-resolved X-ray photoelectron spectroscopy was used to study the exposed chemical groups. The rhBMP-2 binding efficiency and protein state were studied with time-of-flight secondary ion mass spectrometry. Quartz crystal microbalance, atomic force microscopy, and enzyme-linked immunosorbent assay were used to analyze protein–substrate interactions. The effect of the topmost layer was tested on initial cell adhesion and short-term osteogenesis marker expression. The results show the highest expression of selected osteomarkers in cells cultured on the DR-ended layer, while the cellular flattening was rather poor compared to the CS-ended system. rhBMP-2 adhesion was observed only on negatively charged layers. Cell flattening became more prominent in the presence of the protein, even though the osteogenic gene expression decreased.

## 1. Introduction

Various synthetic and natural, biodegradable, and non-biodegradable materials have been used to fabricate bone scaffolds with different methods [1,2]. Multilayer polymeric films are versatile platforms that can be used to coat different charged surfaces [3]. Dip, spray, and spin-coating are typical methods to cover surfaces of interest in order to change their chemistry and physicochemical properties [4]. The adsorption of different materials can alter many crucial parameters of surfaces such as hydrophobicity, chemical composition, charge, roughness, etc. These parameters are crucial for the interactions between the biomaterial surface and cellular membrane [5,6].

Polymeric multilayer surfaces show specific resorbability, surface reactivity, and biocompatibility properties that affect osteoconduction and osteoinduction [7,8]. The most popular synthetic polymers used to fabricate polymeric multilayers are poly(lactic acid) (PLA), poly(glycolic acid) (PGA), poly(lactic-*co*-glycolic acid) (PLGA), poly(ɛ-caprolactone) (PCL), poly(ethylene glycol) (PEG), polyurethane (PUR), etc. [9]. Recently, diazoresin (DR) has attracted attention as a polycation applied in the capillary electrophoresis of proteins [10]. In particular, multilayer capillary coatings composed of DR and poly(styrene sulfonate) (PSS) show good stability and reproducibility. Among natural polymers of special interest are glycosaminoglycans (GAGs), collagen, alginate, chitosan, etc. [9]. The incorporation of growth factors (GFs) into the biomaterial scaffold, whether in the continuous phase or multilayer system, can affect cell behavior and improve osteogenesis. Many bone-derived GFs, such as bone morphogenetic proteins (BMPs), have been isolated from the bone matrix and characterized [11]. In organisms, BMPs exist in free as well as matrix-bound forms [12]. The adsorption of these proteins on the surfaces, their conformation, and activity are dependent on the chemical composition and physical properties of the surface, such as wettability, surface charge, roughness, and topography.

Bone formation requires a contribution of mesenchymal stem cells (MSCs) mediated by various osteoinductive factors, particularly bone morphogenetic protein-2 (BMP-2) signaling [13]. Endogenous BMPs act at low doses (ng or μg) in the tissue; however, supraphysiological levels of exogenous BMPs are necessary at the site of a surgery to achieve osteoinductive properties due to their short half-life [14]. This causes serious dose-related adverse effects due to the high local quantities of high-quality BMPs in clinical applications [11]. To improve cell stimulation with GFs, their effective immobilization and release from materials were studied in the past few years in which physical, covalent, and bioaffinity GF immobilization was achieved [15,16,17]. The immobilization methods exploit different properties of the side groups of the amino acids from the protein. These side groups can be involved in binding the protein to the support through different physicochemical interactions, ranging from reversible physical adsorption, ionic linkages, and affinity binding, to irreversible covalent bonds such as ether, thio-ether, amide or carbamate ones [14,17,18,19].

The flexibility of BMP-2 signaling suggests that the induction of a specific pathway is determined by receptor availability, spatial organization, and association in complexes on the cell membrane [20]. A growing body of evidence suggests that BMP-2 affects actin cytoskeleton organization and takes part in cell adhesion and migration through integrin/BMPR crosstalk. Fourel et al. demonstrated that matrix-bound BMP-2 is able to initiate a β3 integrin-dependent mechanical response in a BMPR-dependent and a Smad phosphorylation-independent manner [21]. Furthermore, it was shown that the interaction of matrix-bound BMP-2 with BMPR causes the initiation of an adhesive and promigratory phenotype through β3 integrin clustering, reorganization of the cytoskeleton through stress fibers, filopodia formation, and an increase in adhesion [21]. To address the spatial regulation of BMPs and the interaction of adhesive molecules with biomimetic materials, Posa et al. employed the surface nanopatterning of covalently immobilized BMP-2 and integrin selective ligands (cyclic-RGD peptides or α_5_β_1_ integrin peptidomimetics) [15]. Their results indicated that BMP-2, both in the free form and immobilized on the surface, causes the assembly of peripheral β_1_ integrin clusters when this integrin subunit is engaged in binding to its ligands.

This paper presents the results of our studies on the interactions of BMP with polymeric ultrathin multilayers composed of DR and CS. The rhBMP-2 was immobilized to the outermost layer in two ways: (1) electrostatically onto previously photocrosslinked multilayers; (2) covalently during the photocrosslinking reaction. Then, 12- or 13-layer systems were prepared, both with and without BMP-2, differing from the topmost layer. Angle-resolved X-ray photoelectron spectroscopy (AR-XPS) was used to analyze the most exposed chemical groups of the multilayers. We have shown the potential of this technique in the analyses of ultra-thin organic systems at 1 nm resolution. Protein interactions with substrates were characterized using the quartz crystal microbalance with energy dissipation (QCM-D), atomic force microscopy (AFM), and Time-of-Flight Secondary Ion Mass Spectrometry (TOF-SIMS) measurements with principal component analysis (PCA). The response of human umbilical cord mesenchymal stem cells (hUC-MSCs) cultured on polymeric layers in the absence and in the presence of BMP-2 was analyzed at different intervals (initial—up to 24 h and short-term—up to seven days). The selected parameters of the cellular response were studied, including cell proliferation and flattening, cytoskeleton organization, and osteogenic marker expression. Importantly, the effect of DR on the expression of different osteomarkers has not yet been described in the literature. The present research utilized a unique combination of techniques allowing physicochemical characterization of the polymeric surfaces and evaluation of the influence of different factors, such as their topography and type of rhBMP-2 immobilization on the biological activity of the whole system.

## 2. Results and Discussion

### 2.1. Characterization of Polymeric Films

The chemical composition and physicochemical parameters of the surface coating are crucial for cell–material interactions. In the first stage, composed multilayer coatings were prepared by dip-coating (i.e., with the LbL method) to produce a sequence of alternate layers of synthetic cationic DR and natural anionic CS. The layers were bound by electrostatic interactions between the cationic diazonium groups of DR and the anionic carboxyl and sulfate groups of CS (see Figure S1 in the SI). Upon UV irradiation, diazonium nitrogen was released and phenyl cations formed, which reacted with sulfate groups, forming C-O covalent bonds. UV–Vis spectra confirmed the formation of covalent bonds as manifested by the decrease in the intensity of the absorption band at 380 nm and the appearance of the band at 290 nm (see Figure S2). The formation of the covalent bonds between layers was also confirmed by the reversal of the surface charge from positive (DR as the outermost) to negative after UV irradiation, as found from ζ-potential measurements. The thickness of the six-bilayer system was around 10 nm in the dry state [22].

**Figure 1:**
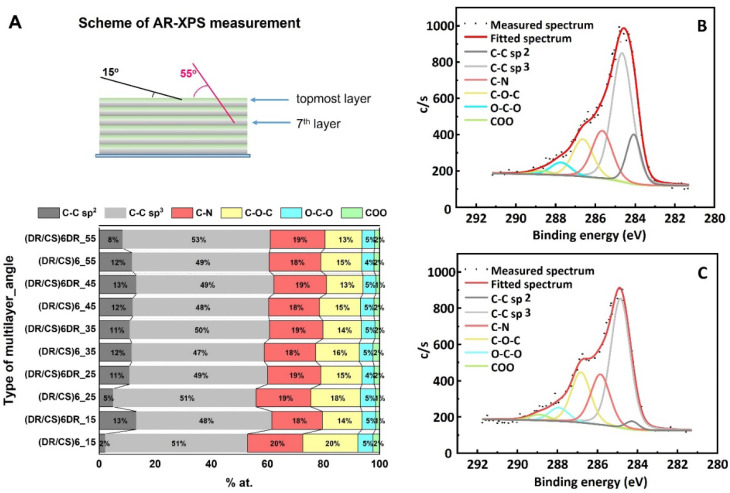
(**A**) Graphical representation of the angle-resolved XPS measurements. The outermost and deeper layers were analyzed at angles of 15 and 55 degrees, respectively. Data were collected using a series of sample tilt angles: 15, 25, 35, 45, and 55 degrees. Contribution of varying carbon chemical states for (DR/CS)_6_ and (DR/CS)_6_DR. XPS-fitted C1s line for photocrosslinked (DR/CS)_6_ (**B**) and photocrosslinked (DR/CS)_6_DR (**C**). The spectra shown were collected at a 15-degree angle.

The composition of the topmost layers in the systems composed of twelve ((DR/CS)_6_) and thirteen ((DR/CS)_6_DR) layers was examined with AR-XPS depth profiling [23,24], In this technique, the emission angle at which the electrons are collected varies, thereby enabling the detection of electrons originating from different depths. At 15 degrees, the topmost layer was analyzed, whereas at 55 degrees, deeper layers were analyzed, corresponding to the depth ranging from ~1.2 nm to 4 nm (graphically illustrated in Figure 1A). Taking into account total multilayer thickness, it could be estimated that the deepest measurements were taken from the 7th layer for the 12-layer system and the 8th layer for the 13-layer system. The total angle-resolved elemental analysis of the photocrosslinked (DR/CS)_6_ and (DR/CS)_6_DR samples is presented in Table S2. Figure 1B,C presents the C 1s spectra of the outermost layer fitted by six lines presenting different carbon chemical states: 284.0 eV (C-C sp^2^), 284.8 eV (C-C sp^3^), 286.6 eV (C-N), 287.7 eV (C-O-C), 287.9 eV (O-C-O), and 288.8 eV (COO) [25]. Figure 1A shows the differences in the carbon state between 12 and 13 layers depending on the depth of the analyzed layer. Spectra collected at 15 degrees revealed significant differences for the C-C sp^2^ contribution (from the carbon atoms of the phenyl groups of DR) to the total carbon amount. Analysis at 15 degrees showed that when DR and CS were the topmost layers, the C-C sp^2^ line constituted 13% and 2%, respectively of the total carbon states, indicating that the penultimate DR layer and the topmost CS layer were well-stratified and practically did not interpenetrate. At 25 degrees, which corresponded to deeper lying layers, the C-C sp^2^ line contribution decreased to 11% within the (DR/CS)_6_DR system, while that of (DR/CS)_6_ increased to 5%, indicating growing interpenetration of both layers. Analysis at 35 and 45 degrees revealed that the deeper layers completely interpenetrated and there were no clear borders between them. Thus, the AR-XPS analysis confirmed that although the composition of the deeper layers was homogenized, the composition of the topmost layers was significantly different for both studied systems, indicating that their interactions with biomolecules and cells could differ significantly [26].

### 2.2. Response of hUC-MSCs Cultured on the Different Topmost Layers

**Figure 2:**
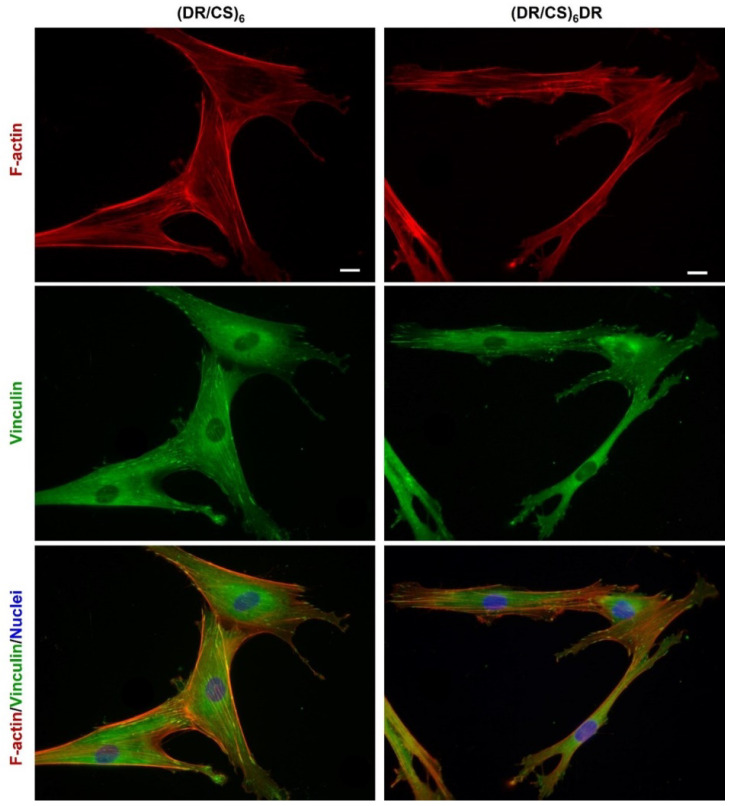
Changes in the actin cytoskeleton architecture in hUC-MSC grown on both types of DR/CS multilayer films. Cells were cultured in a medium supplemented with 2% FBS for 24 h and were then immunostained for vinculin and counterstained with TRITC-phalloidin (F-actin) and DAPI (nuclei). The scale bar represents 25 μm.

**Figure 3:**
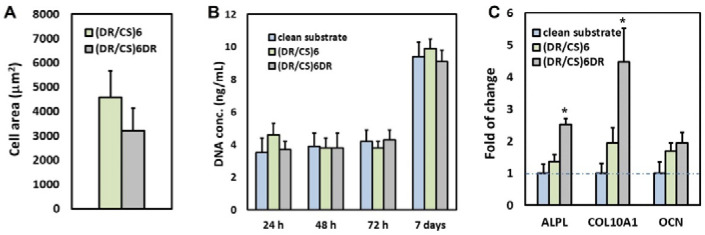
(**A**) Cell area 24 h after seeding. (**B**) DNA concentration of hUC-MSC proliferation evaluated after 24, 48, 72 h, and 7 days of cell-culture on a clean substrate, (DR/CS)_6_ and (DR/CS)_6_DR. (**C**) Expression of osteogenic markers in hUC-MSCs cultured on a clean substrate, (DR/CS)_6_, and (DR/CS)_6_DR for 7 days in vitro. Quantitative real-time PCR analysis of selected osteogenic markers (ALPL–alkaline phosphatase; COL10A1–collagen type 10; OCN–osteocalcin) in hUC-MSCs cultured in DMEM/F12, 2% FBS). Gene expression was normalized to the expression of a housekeeping gene–GADPH, shown as the fold-change compared to gene expression in hUC-MSCs cultured on the clean substrate. *p* values less than 0.05 (*p* < 0.05) were considered statistically significant in comparison to the control and are labeled with an asterisk (*).

In order to analyze the effect of the chemical composition of both the DR and CS topmost layers in the ultrathin multilayer systems obtained, the response of hUC-MSCs cultured on these surfaces was studied. As a control, clean glass substrate was used. The organization of the hUC-MSC cytoskeleton and its proliferation and gene expression were analyzed (Figure 2 and Figure 3).

Adhesion is the first step of cell interaction with a material surface. This crucial stage drives cell proliferation and migration and also induces differentiation if the appropriate conditions are satisfied [6]. Cells respond to microenvironments by adjusting their cytoskeleton organization in the following phases: I–initial adhesion due to complex physicochemical interactions (hydrophobic, coulombic, van der Waals forces); II–flattening due to integrin bonding; and III–full spreading to form a stable focal adhesion [27]. hUC-MSCs were immunofluorescently stained for vinculin and counterstained with TRITC-phalloidin for F-actin to study the effect of the DR or CS topmost layer on focal adhesion and stress fiber organization after 24 h of cell seeding (Figure 2). The images show a significant difference in cell morphology and cytoskeleton organization at the beginning of the cell–material interactions. Cells seeded on (DR/CS)_6_ assumed a more flattened and triangular geometry than those cultured on (DR/CS)_6_DR, which were more elongated. This effect is a consequence of cellular adhesion differences in first cell–material contact. However, microfilament bundles (stress fibers, red) were organized along the base of the cells for both surfaces. Further, cells were anchored to the substrate by creating vinculin-rich focal adhesion complexes (green) at the end of the actin filaments. Cells cultured on DR, as a topmost layer, did not adhere to the substrate uniformly despite the focal contact formation. They formed more microspikes and pseudopodia due to their attempt to move. The mean cell area was calculated for 30 cells for each condition. The area of hUC-MSCs cultured on the CS-terminated system was significantly higher than that cultured on the DR-terminated surface, i.e., around 4600 μm^2^ vs. 3200 μm^2^, respectively (Figure 3A). Cells with higher surface areas interacted more strongly with the substrate, indicating that the CS as a topmost layer stimulated cell adhesion more strongly than DR. It should be noted that the presented polymeric films were ultrathin. Their global exposed potential had similar values: −50 mV for (DR/CS)_6_ and −55 mV for (DR/CS)_6_DR) [22]. The exposure of the saccharide groups in CS enhanced cell adhesion and flattening, while the presence of synthetic DR was not preferable with hUC-MSC during first contact. Chondroitin sulfate, as an extracellular matrix component, enhances material–cell interactions [28]. The CS topmost layers create a more hydrated surface due to the exposed polar groups, thanks to which capturing soluble mediators such as GFs onto the ECM and cell surface ECM is possible in contrast to the DR topmost layers [29].

Cell proliferation on the surface of a material allows its biointegration [30]. Figure 3B presents the cell proliferation after different culture times. After 24 h, the number of cells cultured on CS as a topmost layer was around 1.3-times higher than in the control and the DR-terminated system. However, these differences were not statistically significant. Nevertheless, after 48 and 72 h, the cell number did not increase significantly compared to 24 h of culture. Furthermore, the cell number became similar for both systems, indicating that the effect of the initial interaction diminished after a couple of days (short-term cultivation). After seven days of culture, the hUC-MSC number increased significantly at the same level for each surface. Such multilayers could thus be applied as coatings of bone implants or scaffolds.

To gain deeper insight into the molecular changes induced in hUC-MSCs by the analyzed materials, gene expression analysis was performed using alkaline phosphatase (ALPL), collagen type 10 (COL10A1), and osteocalcin (OCN) osteogenic markers. Gene expression was calculated as the fold-change compared to cells cultured on clean substrates (Figure 3C). The results indicated that the short-term (seven days) expression of relevant genes was higher (1.9-times for ALPL, 2.3-times for COL10A1) for cells cultured on DR-ended layers than for cells cultured on CS-ended layers, while for OCN, the difference was much smaller and statistically insignificant. To the best of our knowledge, no reports in the literature show this effect. The molecular mechanisms linking cell shape to basic cell functions and phenotype maintenance are important and still hardly known. Using a computational model, Vasilevich et al. identified a universal set of genes that regulate the material-induced phenotypical response of human mesenchymal stem cells [31]. They indicated that ALP expression positively correlated with the shape parameters (FormFactor and Minor Axis length), whereas the Extent parameter demonstrated a negative correlation. Thus, a typical ALP-positive cell possesses an elongated, irregular shape with many protrusions. This observation correlates well with our data (Figure 2). Furthermore, these results are consistent with the previous work of Hulshof et. al. [32]. To investigate the relationship between ALP level and cell shape, they employed quantitative measurements of cell morphology from various surfaces with the highest and lowest ALP intensities, respectively. Several cell shape parameters, including the cell solidity, Euler number, and compactness, demonstrated the correlation between the cell shape and ALP expression [32]. Previous studies indicated that surfaces terminated with GAGs such as CS could enhance the attachment, proliferation, and osteogenic differentiation of cells on various scaffolds due to their biocompatibility [33,34]. However, our data also indicate the potential application of DR-terminated coatings in biomedical applications despite the differences in initial cell–material contact. It may be speculated that aromatic rings in DRs affect protein adsorption or stimulate cells’ receptors during short-term cultivation.

### 2.3. Interaction of the Topmost Layers with Immobilized rhBMP-2

**Figure 4:**
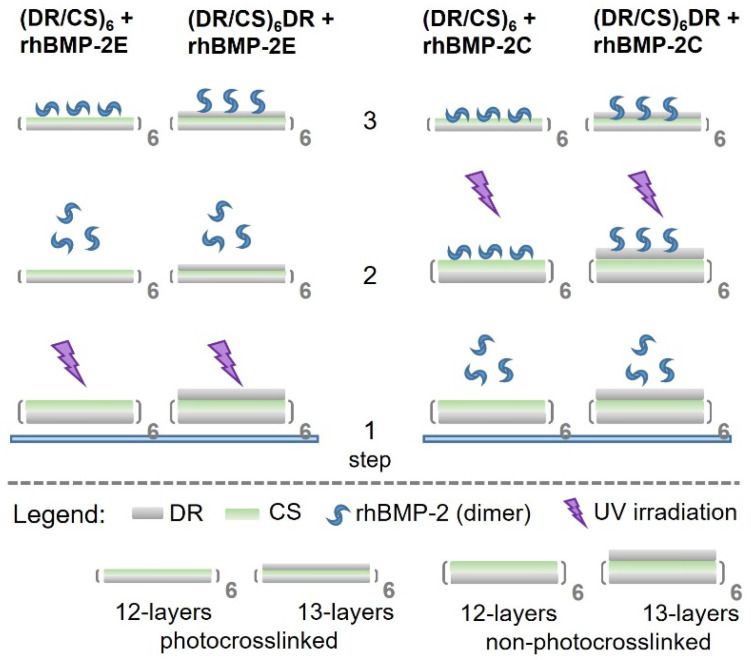
Schematic presentation of the obtained surfaces with preparation steps; E–protein bound electrostatically, C–protein bound covalently. Non-photocrosslinked multilayers are marked as a thicker system, while photocrosslinked layers are thinner.

The interactions of the immobilized rhBMP-2 with the studied surfaces were analyzed. It was expected that the immobilization of rhBMP-2 by the formation of the covalent bonds will also maintain its structure and activity [35]. In order to analyze these interactions, four types of multilayers were prepared (Figure 4, step 1). 

**Figure 5:**
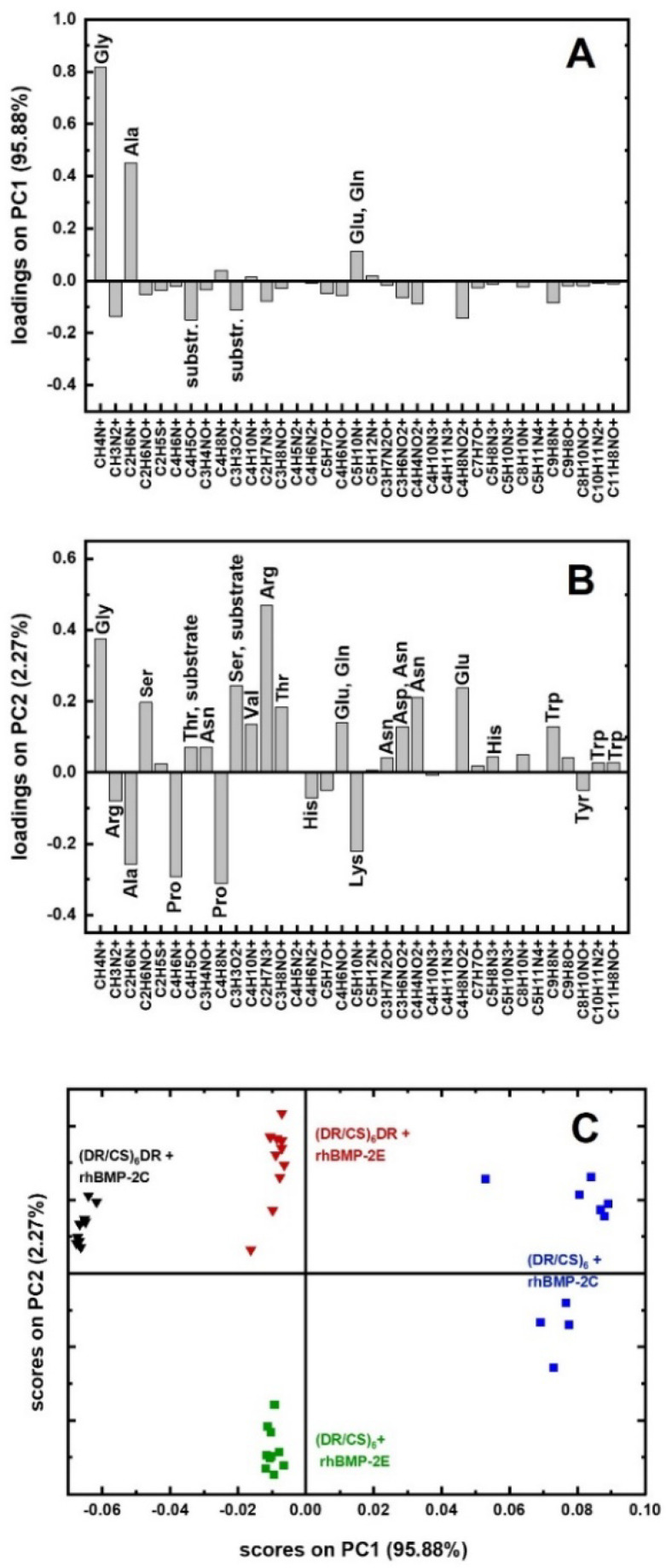
TOF-SIMS analysis of the rhBMP-2 dimer covalently and electrostatically bonded to (DR/CS)_6_ and (DR/CS)_6_DR. (**A**) PC1 is loaded negatively and positively by the mass signals of amino acids and substrates, respectively. (**B**) PC2 is loaded negatively and positively by the mass signals of amino acids exposed to the surface; PC2 is loaded positively by the mass signals of Arg, Ser, and Gly and negatively by the mass signals of Ala, Pro, and Lys, which relates to the different structural state of rhBMP-2. (**C**) Scores plot with four groups of data points, centered in different quadrants of the coordinate system PC1 vs. PC2, corresponding to all samples.

After the photocrosslinking of the multilayers, the rhBMP-2 homodimer was deposited via electrostatic interactions to obtain two systems denoted as (DR/CS)_6_ + rhBMP-2E and (DR/CS)_6_DR + rhBMP-2E (Figure 4, step 2 left side). When the rhBMP-2 homodimer was deposited via electrostatic interactions with the surface followed by photocrosslinking, two other systems were obtained, denoted as (DR/CS)_6_ + rhBMP-2C and (DR/CS)_6_DR + rhBMP-2C (Figure 4, step 2 right side). Thus, four systems were obtained based on the physical (E) and chemical (C) immobilization of rhBMP-2 on both surfaces. TOF-SIMS with multivariate principal component data analysis was used to study the differences in rhBMP-2 interactions within the topmost layers. TOF-SIMS is an attractive technique because of its sensitivity to the outermost region of the surface. The SIMS sampling depth is smaller (~1–2 nm) than the size of the molecules of most proteins, so the differences in the proteins’ state (orientation, conformation) can be examined [36]. This technique evaluated the protein surface coverage in our research and was used to analyse the protein state. The multivariate principal component data analysis allowed the investigation of the protein orientation and conformation regarding exposed amino acids [37]. The main source of the variability of the TOF-SIMS intensities in the analyzed dataset, captured by the first principal component (PC1) at 95.88% (describing most of the total variance), was related to the protein surface coverage (Figure 5A). The second principal component (PC2), capturing 2.27% of the variance, separated samples with different protein states (Figure 5B). The score plot (PC2 vs. PC1) for the developed PCA model obtained for all proteins’ bonded systems revealed the variety of the amounts of rhBMP-2 adsorbed on the studied surfaces (Figure 5C).

TOF-SIMS signals were analyzed for the characteristic contributions of the protein and polymer substrate (see Figure S3). As can be seen, a negligible amount of protein was adsorbed on the DR-ended layer after covalent binding, with no decrease in the substrate-specific signal and very little increase in the protein-specific signal (see Figure S3B–D—right green columns). rhBMP-2 has an isoelectric point around 8.5 and thus it is positively charged below pH 8.5 [38]. That is why a little amount of adsorbed rhBMP-2 could be the effect of electrostatic repulsion between positively charged protein and cationic DR diazo groups present before UV irradiation. The topmost polymeric layers were negatively charged for (DR/CS)_6_-based systems, so rhBMP-2 was adsorbed effectively. Proteins adsorbed on charged interfaces tend to expose oppositely charged outermost regions [35]. Protein covalent immobilization on the CS-terminated system caused a significant increase in the protein-specific signal compared to the polymeric substrate (see Figure S3B–D—left green columns), indicating that the amount of adsorbed rhBMP-2 in the (DR/CS)_6_ + rhBMP-2C system (Figure 5C, blue squares) was highest compared to the other systems. Moreover, as can be seen in Figure 5C, the protein assembled diversified the spatial state via chemical bonding (blue squares). A similar amount of protein was adsorbed on both types of topmost layers via electrostatic interactions (violet columns, see Figure S3), assuming a different and more defined state (Figure 5C, red triangles and green squares). This is a direct effect of the topmost layer on proteins’ arrangement on the polymeric surface. For the CS-ended layer, Ala, Pro, and Lys were exposed to the air, while for the DR-ended layer, Arg, Ser, and Gly were exposed (Figure 5B). Ghaemi at al. have shown TOF-SIMS with PCA for BMP-2 and Collagen type I sample populations, indicating the creation of new surface chemistry when these two proteins were immobilized together or separately [39]. However, there are no reports on BMP-2 structure analysis using this method. Our results correlate well with the observations of cell–material initial contact (Section 2.2), where proteins from culture media could interact with the topmost layer differently and alter cell flattening. The cellular response is more visible when the topmost surfaces significantly differ in charge, roughness, wettability etc. [6,40,41]. As mentioned, the topmost layers were negatively charged in the presented cases, but apart from that, the exposed CS or DR layer affected the proteins’ state and cell flattening. Nevertheless, cells as a dynamic biological system finally adapted to the local microenvironment and started to proliferate at a similar level independently of the exposed ultrathin polyelectrolyte layer. This analysis has not been done previously because DR-terminated polymeric films are usually not chosen for further bioassays. Due to the limited amount of covalently bonded rhBMP-2 on (DR/CS)_6_DR, this system was excluded from further analysis.

### 2.4. Surface-rhBMP-2 Interaction in an Aqueous Environment

**Figure 6:**
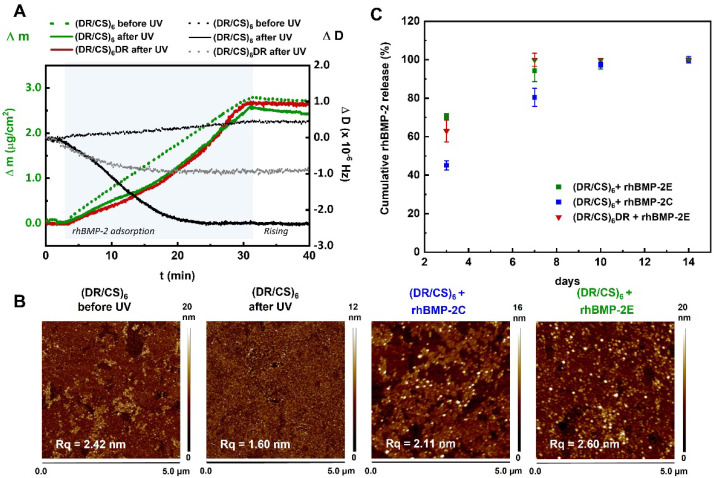
(**A**) rhBMP-2 adsorption/desorption QCM-D measurement using the Au/SiO_2_ sensor coated with (DR/CS)_6_ before photocrosslinking (dotted curves) and (DR/CS)_6_ and (DR/CS)_6_DR after photocrosslinking (solid curves), expressed as the mass shift (green and red curves) and dissipation shift (black and grey curves). rhBMP-2 was used as an aqueous solution of 1 μg/mL, pH 6.2, and flow rate of 0.15 mL/min, adhered for 30 min (blue area). (**B**) Representative topography images of (DR/CS)_6_ multilayers in the liquid state before and after photocrosslinking and after the deposition of rhBMP-2 from 1 μg/mL solution: covalent and electrostatic bonding. Measurements were performed in sterile water at room temperature using tapping mode. The root-mean-square roughness (Rq) values corresponding to each image are shown. (**C**) Release of rhBMP-2 detected by ELISA from surfaces of different types.

It is well known that the substrate changes the microenvironment of the adsorbed protein and alters its stability/activity [35]. The interactions in aqueous environment are crucial for all bioactive molecules, e.g., protein adsorption/desorption and denaturation processes. QCM-D was used to analyze the rhBMP-2 adsorption on selected systems in the aqueous environment. QCM-D uses two independent quantifiable signals, the resonance frequency shift and energy dissipation. In the case of the studied systems, the frequency response is related to the detected mass (Δm) of surface-bound protein, while the dissipation response (ΔD) is related to the viscoelastic properties of the adsorbed rhBMP-2, indicating rigidity (decrease in dissipation) or softness (increase in dissipation) of these films [42]. The analyzed surfaces were the CS-terminated multilayer before and after photocrosslinking and the DR-terminated multilayer after photocrosslinking. The amount of adsorbed rhBMP-2 was similar for all cases: 2.4 μg/cm^2^ for photocrosslinked (DR/CS)_6_ (Figure 6A, solid green line), 2.7 μg/cm^2^ for non-photocrosslinked (DR/CS)_6_ (Figure 6A, dotted green line), and 2.6 μg/cm^2^ for photocrosslinked (DR/CS)_6_DR (Figure 6A, solid red line). The changes in dissipation energy differed between the photocrosslinked and non-photocrosslinked surfaces (Figure 6A, black and grey lines). The CS-terminated system before UV exposure behaved as a typical polymer assembly, and ΔD increased with a mass up-take (up to 0.5 × 10^−6^) [43]. On the other hand, for both photocrosslinked systems, ΔD decreased to negative values due to rhBMP-2 adsorption. This suggests that the protein adsorption on the CS-terminated surface made the surface stiffer than the adsorption on the DR-terminated multilayer. This difference in final surface rigidity could correlate with the assembled rhBMP-2 state analyzed from TOF-SIMS (Figure 5, red triangles and green squares). The usefulness of combining these two techniques for protein–material interaction and orientation analyses has been reported [44,45]. They are complementary, although TOF-SIMS measurements are carried out under ultra-high vacuum, whereas QCM-D provides data collected in an aqueous environment.

The atomic force microscopy showed the topography of a 12-layer system before and after UV irradiation (Figure 6B, two left images). The images indicate that the photocrosslinking decreased the roughness from 2.42 nm to 1.6 nm. This result is in good agreement with literature reports [46]. However, together with reducing the roughness, crosslinking increases the stiffness. This is the crucial result of why there was a slight increase in ΔD observed for rhBMP-2 adsorption on non-photocrosslinked (DR/CS)_6_ in contrast to the previously photocrosslinked multilayer, for which a significant decrease in ΔD was observed. It can be concluded that the adsorption of rhBMP-2 in the rigid (crosslinked) system caused an increase in softness, while for the softer (non-crosslinked) system, the rhBMP-2 adsorption caused an increase in rigidity.

Figure 6B shows the atomic force microscopy images of multilayers with covalently and electrostatically bonded rhBMP-2 represented by white spots. The presence of protein increased the substrate roughness. Due to the limited sensitivity, the BCA for the total protein content was not applicable for these systems.

The rhBMP-2 release profile was analyzed with the enzyme-linked immunosorbent assay (ELISA). According to the assay manufacturer, it detects rhBMP-2 only in the biologically active form. rhBMP-2 activity was retained, as could be expected from previous studies, which have shown that neither photo-immobilization nor covalent bonding caused rhBMP-2 denaturation [47,48]. The release of rhBMP-2 was analyzed on day 3, 7, 10, and 14 (Figure 6C). Such time steps were chosen according to the standard protocol of culture medium exchange, which was done twice per week. The highest amount of rhBMP-2 (70%) was released until the third day from (DR/CS)_6_ + rhBMP-2E, while from (DR/CS)_6_DR + rhBMP-2E, around 63% of the protein was released. Such a difference could be an effect of the protein adsorption state on the discussed systems. The smallest amount of protein (45%) was released from (DR/CS)_6_ with covalent binding of the protein. It was expected that a burst release occurred within the first days or even hours [49]. On day 7, the electrostatically bonded protein was practically completely released into the medium (PBS) (>95%). Protein release from (DR/CS)_6_ + rhBMP-2C on day 7 was around 80%. On days 10 and 14, rhBMP-2 was completely released from all systems. The observed protein released from the covalently bonded system could be the effect of surface degradation.

### 2.5. hUC-MSCs Response to rhBMP-2 Immobilized on the Surface

**Figure 7:**
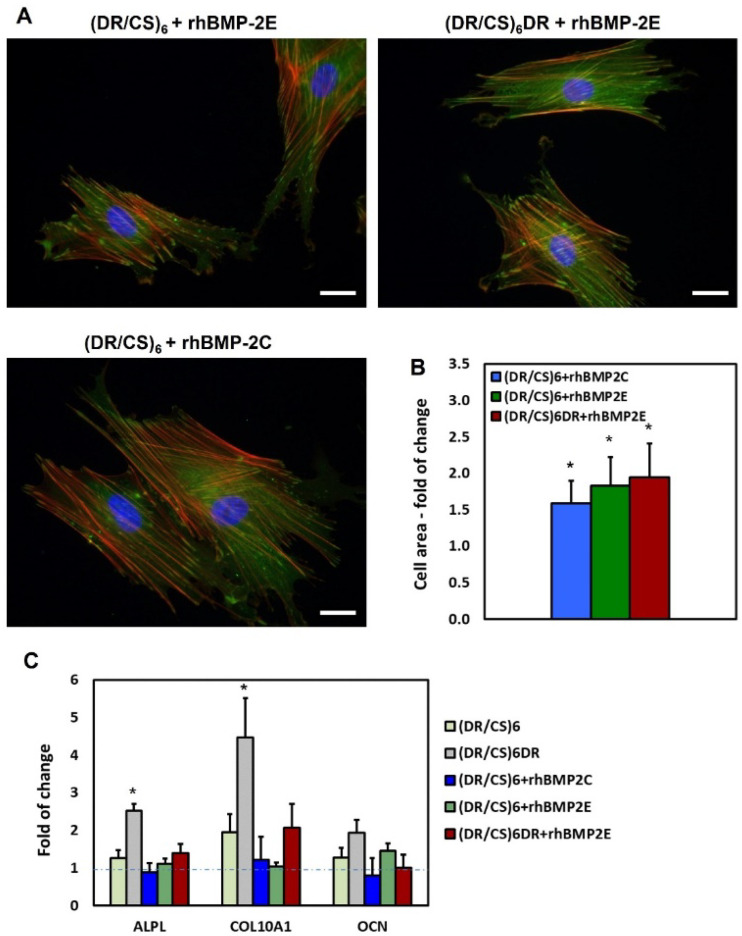
(**A**) Changes in the actin cytoskeleton architecture of hUC-MSCs cultured on selected surfaces with immobilized rhBMP-2. Cells were cultured in a medium supplemented with 2% FBS for 24 h and were then immunostained for vinculin and counterstained with TRITC-phalloidin (F-actin) and DAPI (nuclei). The scale bar represents 25 μm. (**B**) Cell area after 24 h of seeding measured for 30 cells for each condition, shown as the fold-change vs. appropriate pure polymeric systems (controls). (**C**) Expression of osteogenic markers in hUC-MSCs cultured on selected surfaces for seven days. Quantitative real-time PCR analysis of selected osteogenic markers (ALPL–alkaline phosphatase; COL10A1–collagen type 10; OCN–osteocalcin) in hUC-MSCs cultured in DMEM/F12, 2% FBS. Gene expression was normalized to the expression of a housekeeping gene–GADPH, shown as the fold-change compared to gene expression in hUC-MSC cultured on the clean substrate–glass coverslips (blue line). *p* values less than 0.05 (*p* < 0.05) were considered statistically significant in comparison to the control and are labeled by an asterisk (*).

Protein immobilization is a common approach to changing the microenvironment for cells [17]. Therefore, it is essential to recognize the protein’s structural rearrangement upon immobilization, depending on the choice of immobilization method and if it affects their biological activity. The best way to analyze the protein activity is to investigate if it causes changes in the cellular response. Therefore, we studied the response of hUC-MSCs to rhBMP-2 immobilization in the abovementioned systems. As shown in Figure 7A, protein immobilization significantly affected cell flattening. hUC-MSCs cultured on surfaces with rhBMP-2 immobilized formed dense and prominent microfilament bundles (actin stress fibers, red) and vinculin-rich focal adhesion complexes (green). Cells seeded on selected surfaces with deposited rhBMP-2 had an area in the range of 1.5- to 2-times statistically significantly larger than that of appropriate control cells, i.e., cells cultured on polymeric multilayers without immobilized rhBMP-2 (Figure 7B). Recent studies have shown that this improved adhesion may result from the interplay between integrins and BMP receptors [16,50]. Mesa-Restrepo et al. [51] reported that the presence of BMP-2 on Ti discs plays an important role in cellular adhesion and spreading of C2C12 cells by increasing of filopodia number per cell and vinculin expression. It was shown that BMP-2 enhances the formation of focal adhesions and stress fibers in osteoblasts by increasing α5 and β1 integrin expression and also triggers cell migration [52]. The synergistic effect of BMP-2 on cell adhesion and mechanotransduction is possible only when rhBMP-2 is immobilized on the material surface, i.e., not dissolved in the medium [53].

After seven days of cell culture, the expression of selected genes was analyzed (Figure 7C). A significant decrease in gene expression, i.e., 1.8-fold for ALPL, 2.2-fold for COL10A1, and 1.9-fold for OCN, was observed on the (DR/CS)_6_DR+rhBMP2E surface compared to (DR/CS)_6_DR (red and grey bars). In the case of (DR/CS)_6_ systems with covalent and electrostatic rhBMP-2 binding, gene expression decreased in a range of 1.1- to 1.9-fold compared to the polymeric multilayer system. The changes in ALPL and COL10A1 expression were statistically significant.

An interesting correlation was observed: the gene expression decreased with increasing cell area. These results are crucial from the cell differentiation point of view, especially in understanding the complex interplay of the protein–biomaterial interactions and their further effect on stem cells. Based on the obtained results we speculate that this is related to the interaction of rhBMP-2 and integrins. rhBMP-2 might associate with integrins in a signaling platform that would activate both BMP2R and integrin receptors or activate integrin receptors through binding to the BMP-2 receptor. The second problem is the correlation of gene expression with cell morphology. As described in Section 2.2, the cell shape corelated with ALPL (early osteogenesis marker) expression. The higher the ALPL expression, the more elongated and irregular the morphology of the cells [32]. Collagen, as cell adhesion protein, is commonly used as a cellular support on various biomaterials. Surprisingly, our data also showed the decrease in COL10A1 (late osteogenesis marker) expression after rhBMP-2 immobilization, together with better cell flattening. It is impossible to unambiguously indicate the mechanism that drove all of these cellular responses. According to obtained results, there is a need to step back in the field of rhBMP-2 studies and analyze the effect of small structural changes of a substrate on protein activity rather than overload the material with supraphysiological dosages of this active protein.

## 3. Material and Methods

### 3.1. Materials

Chondroitin sulfate A sodium salt from bovine trachea (CS) and 4-diazodiphenylamine sulfate were purchased from Merck (Darmstadt, Germany). Sodium chloride, paraformaldehyde, zinc chloride, hydrogen peroxide 30%, and sulphuric acid 96% were received from POCh (Gliwice, Poland). Recombinant Human/Mouse/Rat BMP-2 (rhBMP-2, CHO derived) was purchased from Peprotech (Cranbury, USA). All reagents were used as received. Water was distilled twice and deionized using a Simplicity Millipore Water Purification System. QCM sensors (14 mm in diameter, 5 MHz, Cr/Au/SiO_2_) were purchased from QuartzPro (Stockholm, Sweden). Silica wafers (11 mm × 11 mm) were received from Si-Mat (Kaufering, Germany). Glass coverslips (*Ø* = 15 mm) were bought from Mercateo (Krakow, Poland).

### 3.2. Diazoresin Synthesis and Multilayer Preparation

Diazonium resin (DR) was obtained in the reaction of 4-diazodiphenylamine sulfate with paraformaldehyde according to a previously reported procedure [54]. The layers were fabricated using 2 mg/mL diazoresin solutions in deionized water and 1 mg/mL chondroitin sulfate (CS) solution in 0.1 M NaCl. The multilayer polymeric films were prepared as previously reported (see the Supporting Information, SI) [22]. Multilayers composed of six DR/CS bilayers ((DR/CS)_6_), i.e., with the topmost anionic CS layer, as well as (DR/CS)_6_DR, i.e., with the topmost cationic DR layer, were prepared.

### 3.3. X-ray Photoelectron Spectroscopy Measurements

Angle-Resolved XPS (AR-XPS) spectra were recorded on a PHI 5000 VersaProbe II (ULVACPHI, Chigasaki, Japan) system using a micro-focused (100 μm, 25 W) Al Kα X-ray beam with photoelectron take-off angles of 15, 25, 35, 45, and 55 degrees. A dual-beam charge neutralizer was used to compensate for the charge-up effect. High-resolution spectra were collected with an analyzer pass energy of 46.95 eV. The operating pressure in the analytical chamber was less than 5 × 10^−7^ Pa, and a sample area of 300 × 300 μm was scanned. All XPS peaks were referenced to the neutral (C–C) carbon C 1s peak at a binding energy of 284.8 eV. MultiPak v. 9.9.0.8 software, provided by the manufacturer, was used to analyze the data. Spectral backgrounds were subtracted using the Shirley method.

### 3.4. Culture of hUC-MSCs

The human umbilical cord mesenchymal stem cell line (hUC-MSCs) was bought from PromoCell. Cells were cultured in DMEM/F12 medium (Merck, Germany) supplemented with 10% heat-inactivated FBS (Merck, Germany), 100 IU/mL penicillin, and 10 μg/mL streptomycin at 37 °C, 5% CO_2,_ and 95% humidity.

### 3.5. Cell Flattening, Focal Adhesion, and Actin Cytoskeleton Analysis

The cell area was analyzed for 30 cells for each condition after 24 h of culture using ImageJ (version 1.53e). For cytoskeleton analysis, hUC-MSCs were plated at an initial cell density of 1.5 × 10^4^ cells/well onto the glass coverslips, which were clean (control) or covered by different PEM films. The cells were cultured for 24 h and fixed in 3.7% formaldehyde, solubilized with 0.1% Triton X-100, immunostained with mouse monoclonal anti-human vinculin IgG (Merck, Darmstadt, Germany) and Alexa Fluor 488-conjugated goat anti-mouse IgG-clone A11001 (Merck, Darmstadt, Germany), and counterstained with TRITC-phalloidin (Merck, Germany). Nuclei were stained with DAPI (Merck, Germany). The specimens were mounted onto coverslips with poly(vinyl alcohol) (Dako Fluorescent Mounting Medium, Agilent Technologies, Santa Clara, CA, USA), visualized under a fluorescence inverted microscope (Axio Vert.A1, Zeiss, Dresden, Germany), and analyzed using ZEN 2.3 (Zeiss) software.

### 3.6. Proliferation Test

The influence of polymeric multilayers on the growth and propagation of hUC-MSCs was evaluated using the commercially available CyQUANT^®^ Cell Proliferation Kit (Thermo Fisher Scientific, Waltham, MA, USA), which allows determination of the cell number based on the fluorescence enhancement of the dye upon binding to nucleic acid. Briefly, hUC-MSCs were seeded at 1.5 × 10^4^ cells/well density in DMEM/F12 medium with 10% FBS on a 24-well culture plate with glass coverslips covered with (DR/CS)_6_ and (DR/CS)_6_DR. A clean glass coverslip was used as a control (pure substrate). After 24 h, 48 h, 72 h, and 7 days, the quantity of nucleic acids was determined according to the kit manufacturer’s protocol. The fluorescence of samples was measured using a fluorescence microplate reader (Tecan, Mannedorf, Switzerland) at λ_exc_ = 480 nm and λ_em_ = 520 nm. The final DNA concentration was analyzed according to the prepared DNA standard curve for the CyQUANT^®^ Cell Proliferation Assay. The experiment was performed 3 times in triplicate.

### 3.7. Induction of Osteogenic Differentiation of hUC-MSCs Cultured on Multilayer Polymeric Films

hUC-MSCs were seeded on multilayer polymeric films at 1.5 × 10^4^ cells/well density in DMEM/F12 medium with 10% FBS on a 24-well culture plate with glass coverslips covered with (DR/CS)_6_ and (DR/CS)_6_DR. Clean glass coverslips were used as a control. After 6 h, when cells adhered to all variants of the culture surface, the medium was changed to DMEM/F12 supplemented with 2% FBS. The medium was exchanged every 3 days. After 7 days, the total cellular RNA was isolated by a Gene MATRIX Universal RNA Purification Kit (Eurx Ltd., Gdansk, Poland) according to the manufacturer’s protocol. Next, reverse transcription was performed using 1 μg RNA and NG dART RTP CR kit (Eurx Ltd., Gdansk, Poland) and the C1000 Touch Thermal Cycler (Bio-Rad Laboratories, Hercules, USA). To analyze the levels of selected genes activated during osteogenesis, qRT-PCR analysis was performed with the QuantStudio 6 Flex Real-Time PCR System (Thermo Fisher, Waltman, MA, USA), SYBR Green Master Mix (Eurx Ltd., Gdansk, Poland), 100 ng cDNA and 7.5 μM of specific primer to detect alkaline phosphatase (ALPL), collagen type 10 (COL10A1), and osteocalcin (OCN) (Merck, Darmstadt, Germany). The sequences of the utilized primers are included in Table S1 (see the SI). The mRNA expression level was normalized to the housekeeping gene GAPDH. The real-time PCR conditions were as follows: 95 °C for 15 min, 40 cycles of denaturation (15 s, 94 °C), annealing (30 s, 55 °C), and extension (30 s, 72 °C).

### 3.8. Protein Immobilization

The rhBMP-2 was deposited onto polymeric surfaces with the topmost layer composed either of DR or CS. The protein was deposited both before and after the photocrosslinking of multilayers (Figure 4—step 1, left side and step 2, right side), so the protein was bound to the surface covalently (C) and electrostatically (E), respectively. Thus, four systems were obtained and studied: (DR/CS)_6_ + rhBMP-2C, (DR/CS)_6_DR + rhBMP-2C, (DR/CS)_6_ + rhBMP-2E, and (DR/CS)_6_DR + rhBMP-2E (Figure 4). The substrates were incubated for 15 min with 1.00 μg/mL rhBMP-2 aqueous solution (pH 6.2). Incubation was carried out in air saturated with water vapor. Then, the substrates were gently washed with water.

### 3.9. Time-of-Flight Secondary Ion Mass Spectrometry (TOF-SIMS) Measurements

TOF-SIMS analysis was performed using the TOF.SIMS 5 (ION-TOF GmbH) instrument. As primary ions, the Bi_3_^+^ ion clusters produced by a 30 keV liquid metal ion gun were applied. An ion dose density of about 10^12^ ion/cm^2^ and a current of about 0.5 pA were applied in all measurements to ensure static mode conditions. A low-energy electron flood gun was used for charge compensation. Positive and negative ions’ high mass resolution TOF-SIMS spectra were acquired from ten non-overlapping 100 μm × 100 μm areas of each sample with an applied resolution of 128 × 128 points. Mass calibration was performed with H^+^, H_2_^+^, CH^+^, C_2_H_2_^+^, and C_4_H_5_^+^ and H^−^, C^−^, C_2_^−^, C_3_^−^, and C_4_^−^ peaks for positive and negative spectra, respectively. A minimal mass resolution (m/∆m) > 8000 at C_4_H_5_^+^ and (m/∆m) > 6000 at C_4_^−^ was obtained.

### 3.10. Multivariate TOF-SIMS Analysis with PCA

Principal component analysis was performed for the positive TOF-SIMS spectra using the PLS Toolbox (Eigenvector Research, Manson, WA, USA) for MATLAB (MathWorks, Inc., Natick, MA, USA). The intensities of selected peaks from each spectrum were normalized to the sum of selected peaks and were mean-centered before running the PCA.

### 3.11. QCM-D Assembly Study

A Q-Sense quartz crystal microbalance with a dissipation monitoring system (QCM-D, Q-Sense, Gothenburg, Sweden) was used to monitor the deposition of rhBMP-2 onto sensors coated with the PEM. Sensor surfaces were deposited with polymers according to the procedure described in the SI. The QCM measurements were carried out according to the standard method described in the literature [55]. Initially, a stable baseline for deionized water was obtained. After stabilization of a baseline, an rhBMP-2 solution of a controlled concentration was pumped through the cell at a fixed flow rate of 0.15 mL/min for 30 min. Subsequently, pure water was flushed through the cell to study protein molecule desorption. The adsorbed rhBMP-2 mass per unit area (coverage) was calculated using Sauerbrey’s equation [56]:

(1) $$\Delta m=-C_{Q\;}\frac{\Delta f}{n}$$

where Δ*m* is the mass change, Δ*f* is the frequency change, *n* is the overtone number, and *C_Q_* is the mass (coverage) sensitivity constant equal to 0.177 mg/(m^2^ × Hz) for the 5 MHz AT-cut quartz sensor [56].

### 3.12. Atomic Force Microscopy (AFM) Measurements

AFM analysis of the substrates was conducted using a Bruker BioScope Catalyst (Bruker Nano Surfaces, Santa Barbara, CA, USA) coupled with an inverted optical microscope (Axio Observer Z1 from Zeiss, Oberkochen, Germany). A low thermal energy probe was used (FastSscan-Dx, Bruker Probes) with a nominal tip radius of 8 nm and with a 0.25 N/m spring constant. The resonant frequency of the probe in liquid was approximately 110 kHz. Images were obtained in tapping mode at a typical line frequency of 1 Hz. Measurements were performed in sterile water at room temperature only for the following selected 12-layer systems: 1–(DR/CS)_6_ non-photocrosslinked; 2–(DR/CS)_6_ photocrosslinked; 3–(DR/CS)_6_ + rhBMP-2E; 4–(DR/CS)_6_ + rhBMP-2C. The detailed description of AFM analysis can be found elsewhere [57].

### 3.13. In Vitro Release of the rhBMP-2–ELISA Assay

All substrates with rhBMP-2 adsorbed on them were incubated at 37 °C in a 5% CO_2_ atmosphere. At each time point (3, 7, 10, 14 days), an aqueous solution with rhBMP-2 released from the substrates was collected and stored at −80 °C until analysis. Then, 1.0 mL of fresh deionized water was added to each well for further testing of rhBMP-2 release. The concentrations of rhBMP-2 were measured by using a commercial competitive ELISA kit (intra assay CV < 8%, inter-assay CV < 10%, ELISAGenie, UK). The optical density (OD) was determined spectrophotometrically at 450 nm, using an ELISA plate reader Infinite M Nano (Tecan, Mannedorf, Switzerland). The experiments were carried out in triplicate.

### 3.14. Statistical Analysis

Statistical analysis was performed using GraphPad Prism software. One-way ANOVA and Bonferroni (post-hoc test) tests were applied. *p* values less than 0.05 (*p* < 0.05) were considered statistically significant and were labeled with an asterisk (*).

## 4. Conclusions

There is a plethora of studies regarding rhBMP-2 interactions with bone scaffolds and cells. The main limitation of rhBMP-2-based therapy is dosage-related adverse effects. Therefore, new methods for minimizing the amount of used rhBMP-2 should be developed. In our study, we took this limiting/critical factor into consideration and examined the effect of different surfaces (topmost layers) on rhBMP-2 immobilization and stem cell response to these surfaces. For the first time, the potential of combining the very sophisticated techniques, AR-XPS and TOF-SIMS with PCA in ultra-thin multilayer systems’ analysis, was shown. So far, they were commonly used for well-defined structures. These results gave us important information that the topmost layer was well-stratified (even ultrathin) and that the biological interaction depends on the exposed functional groups of this layer. rhBMP-2 was effectively deposited only on the negatively charged topmost layers. The presented results revealed differences between the protein state after electrostatic binding on DR-terminated and CS-terminated layers. Adsorption of rhBMP-2 caused a more significant increase in the softness of the CS-terminated system than the DR-terminated one. In the case of a CS-terminated and non-photocrosslinked system, rhBMP-2 adsorption stiffens the multilayer (changes in ΔD). In this system, according to TOF-SIMS analyses, protein was adsorbed at the highest amount and assumed a diverse state. The release of covalently bound protein was observed up to seven days (ELISA). These results could help in choosing the optimal system providing the best conditions for protein adsorption. There was a correlation between the effect of the topmost layer and the stem cell shape and osteomarker expression. Surprisingly, the immobilization of rhBMP-2 (electrostatically or covalently) enhanced cell flattening while decreasing osteomarker expression. The rhBMP-2 activity was preserved upon immobilization and resulted in enhanced cell adhesion. The expression of selected genes was higher in the absence of immobilized rhBMP-2 in the (DR/CS)_6_ or (DR/CS)_6_DR system. More research should be done to analyze this effect, especially the role of small structural changes of a substrate at the chemical and physicochemical levels on protein activity, which would prevent the application of supraphysiological dosages of this active protein. Moreover, it seems that the best approach would be initial cell stimulation by rhBMP-2 immobilized on the substrate followed by the continuous stimulation from the bulk rhBMP-2.

## Supplementary Materials

The following supporting information can be downloaded at: https://www.mdpi.com/article/10.3390/ijms23169287/s1.
