# Supplementary material for: The Effect of the Topmost Layer and the Type of Bone Morphogenetic Protein-2 Immobilization on the Mesenchymal Stem Cell Response
Source: Int J Mol Sci. 2022 Aug 18;23(16):9287. doi: 10.3390/ijms23169287 (PMC9408842; doi:10.3390/ijms23169287)
Supplement: Supplementary file 1 [file ijms-23-09287-s001.zip › ijms-1872868-supplementary.pdf]

Supporting Information

The Effect of the Topmost Layer and the Type of Bone Morphogenetic Protein-2 Immobilization on the Mesenchymal Stem Cell Response

Magdalena Wytrwal-Sarna, Małgorzata Sekuła-Stryjewska, Agata Pomorska, Ewa Ocłoń, Katarzyna Gajos, Michal Sarna, Ewa Zuba-Surma, Andrzej Bernasik, Krzysztof Szczubiałka

1. Multilayer preparation

Before the multilayers deposition, all substrates (silicon wafers, glass coverslips or quartz substrates) were cleaned by so-called "piranha" solution (H_2SO_5) (i.e., a mixture of 30% solution of H_2O_2 and concentrated H_2SO_4 at 1:3 ratio). Because the substrates were negatively charged, the first deposited layer was polycation. After 5 mins in a sonic bath, substrates were washed by deionized water three times. Next, the polyanion solution was used and placed in a sonic bath. All steps were repeated to obtain the desired systems composed of 12- or 13-deposited layers, respectively as $(DR/CS)_6$ or $(DR/CS)_6DR$. Subsequently, multilayer systems were stabilized using the photocrosslinking method (irradiation by UV lamp λ = 350 nm for 3 mins). Photoreactions between functional groups are presented in Figure S1. The confirmation of photoreaction is the shifting of maximum absorption of DR from 380 nm to 290 nm (Figure S2).

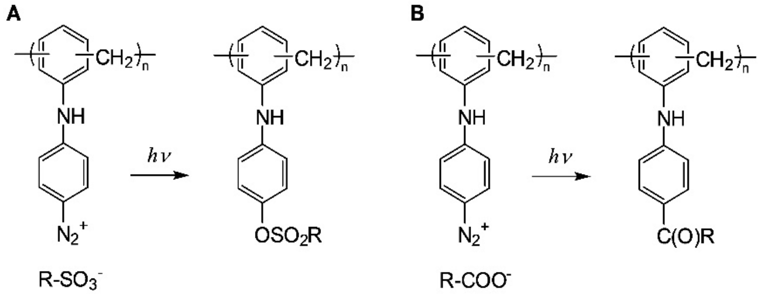

Figure S1 Photocrosslinking reaction between diazonium groups of DR and carboxyl – A and sulfonic groups – B of chondroitin sulfate.

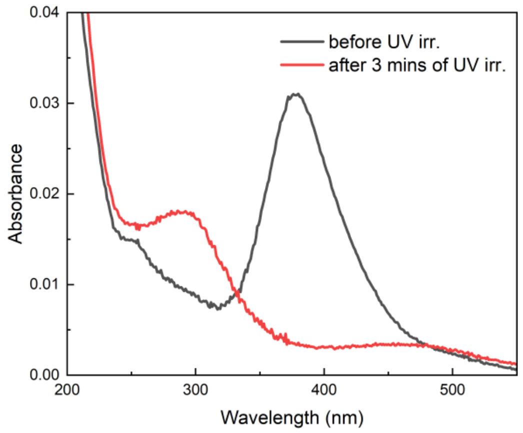

Figure S2 UV-Vis spectra of $(DR/CS)_6$ non-photocrosslinked - before UV irradiation (black line) and photocrosslinked - after 3 minutes of UV irradiation (red line).

2. Real-time PCR

Table S1 Primer list and primer sequences that were used in quantitative real-time PCR analysis of transcripts involved in osteogenic differentiation. F - forward sequence; R – reverse sequence.

Primer name	Sequence
Human GAPDH -F	CTTTTGCGTCGCCAG
Human GAPDH -R	TTGATGGCAACAATATCCAC
Human ALPL -F	CTATCCTGGCTCCGTGCTCC
Human ALPL -R	TTAACTGATGTTCCAATCCTGCG
Human COL10A1 -F	GCTAGTATCCTTGAACTTGG
Human COL10A1 -R	CCTTTACTCTTTATGGTGTAGG
Human OCN -F	GACTGTGACGAGTTGGCTGA
Human OCN -R	CTGGAGAGGAGCAGAACTGG

3. AR-XPS measurements - atomic composition

Table S2 Total XPS elemental analysis of photocrosslinked $(DR/CS)_6$ and $(DR/CS)_6DR$ samples measured with angled resolution.

**Angle (°)**	**$(DR/CS)_6$**				**$(DR/CS)_6DR$**			
	**C**	**N**	**O**	**S**	**C**	**N**	**O**	**S**
	**% at.**				**% at.**			
15	70.4	6.5	21.7	1.5	73.4	7.4	17.9	1.3
25	68.8	6.7	23.5	1.0	71.2	7.1	20.2	1.5
35	66.7	6.8	24.9	1.7	70.5	7.5	21.2	0.9
45	65.8	7.2	25.8	1.1	68.9	7.3	22.2	1.7
55	64.4	7.8	27.0	0.9	69.9	7.7	21.9	0.5
75	63.4	7.6	27.6	1.5	67.0	8.1	23.9	1.1

4. TOF-SIMS measurements

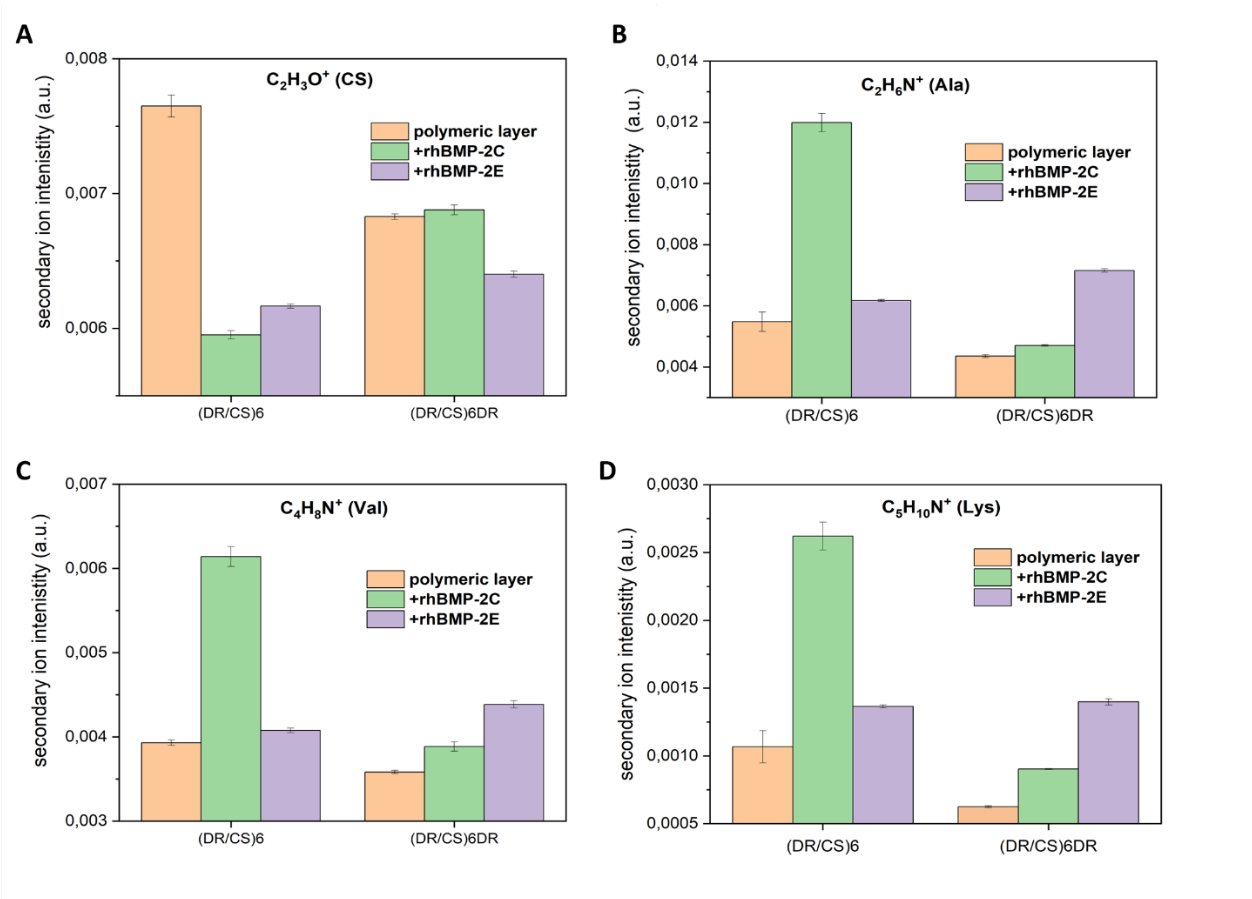

Figure S3 Intensity of selected secondary ions typical for: A – acetyl group of CS; B – alanine; C – valine; D – lysine from rhBMP-2. Six analyzed layouts: bare $(DR/CS)_6$, covalent binded protein - $(DR/CS)_6$-rhBMP-2C; electrostatic binded protein $(DR/CS)_6$-rhBMP-2E; bare $(DR/CS)_6$DR, covalent binded protein - $(DR/CS)_6$DR-rhBMP-2C; electrostatic binded protein $(DR/CS)_6$DR-rhBMP-2E.
